# The usefulness of S100 protein as a predictive factor in patients with hanging injury

**DOI:** 10.1186/2197-425X-3-S1-A855

**Published:** 2015-10-01

**Authors:** E-J Park, J-P Cho, S-C Kim

**Affiliations:** Ajou University Hospital, Suwon, Korea Republic of Korea

## Introduction

Hanging is one of the leading method of suicide in South Korea [1]. Brain injury due to hanging leads to the high mortality rate and the severe neurological sequelae [2, 3]. Serum S100 for predicting the brain injury in hanging injury is not evaluated.

## Objectives

The aim of this study is to review the characteristics and the prognosis of hanging patients and to determine the usefulness of S100 as a predictive factor of prognosis.

## Methods

A single center, retrospective study was performed from January 2011 to December 2014. Total of 103 patients who were over 18 years of age and who visited emergency department (ED) with hanging injuries were enrolled. Individual characteristics, information related to hanging and cardiac arrest were collected. Laboratory test, imaging test, neurological sequelae or the Cerebral Performance Category (CPC) score were collected in 71 resuscitated patients.

## Results

Of all the patients, 57(55.3%) patients were male and 97(94.2%) patients committed suicide by hanging. 61(59.2%) patients visited ED with cardiac arrest. Median duration of hanging injury was 17.0 minutes(IQR 44.0). Mortality rate was 71.8% and the severe neurological sequelae were observed in 11(15.5%) patients. In arrest patients, all the survived patients showed CPC score as 4. Although 17(40.5%) had the initial mental status as stupor or coma in non-arrest patients, 1(2.4%) had the severe neurological sequela. Among the resuscitated patients, comatos mental status (p < 0.00) at admission, the absence of pupil light reflex (p < 0.00) at admission and diffuse swelling in brain computed tomography (CT)(p < 0.00) had tendency related to high mortality rate. Serum S100 levels were elevated and related to the poor CPC score or the severe neurological sequelae(p < 0.00). Pupil light reflex at admission only had tendency related to the neurological sequelae in non-arrest patients, whereas mental status at admission and diffuse swelling in brain CT were not related to the neurological sequelae. The elevated level of serum S100 was not related to the CPC score nor the neurological sequelae in arrest patients or non-arrest patients, respectively.

## Conclusions

The prognosis of hanging patients were related to the presence of pupil light reflex irrespective of the presence of cardiac arrest. Although serum S100 level was elevated in hanging patients, the usefulness in the predicting prognosis is not sufficient.
